# Lessons from the functional characterization of lncRNAs: introduction to mammalian genome special issue

**DOI:** 10.1007/s00335-022-09956-x

**Published:** 2022-05-09

**Authors:** Lynne Maquat, Rory Johnson, Saba Valadkhan, Piero Carninci

**Affiliations:** 1 Department of Biochemistry and Biophysics, School of Medicine and Dentistry, University of Rochester Medical Center, 601 Elmwood Ave, Rochester, NY 14642 USA; 2 Center for RNA Biology, University of Rochester, 601 Elmwood Ave, Rochester, NY 14642 USA; 3 School of Biology and Environmental Science, University College Dublin, Dublin, Republic of Ireland; 4 Department of Molecular Biology and Microbiology and Case Comprehensive Cancer Center, Case Western Reserve University, Cleveland, OH 44106 USA; 5 RIKEN Center for Integrative Medical Sciences (IMS), Yokohama, Kanagawa 230-0045 Japan; 6 Human Technopole, Via Rita Levi Montalcini 1, 20157 Milan, Italy

Some of us may remember betting on the number of mammalian genes and then being surprised to learn, some 20 years ago, that genomes encode only approximately 20,000 protein-coding genes. Offering additional surprises, transcriptome studies including ENCODE and FANTOM revealed that a large part of mammalian genomes is transcribed into long non-coding RNAs (lncRNAs), which were defined later as RNAs > 200 nucleotides with no apparent protein-coding potential. Indeed, from a tally of ~ 3000 in the early 2000s, the present universe of known human lncRNAs has passed 100,000 and continues to grow. Their relatively low expression level and lower evolutionary conservation across species have sparked emotional debates over their function, or lack thereof.

LncRNAs are indeed less conserved than protein-coding genes. However, the evolutionary constraints on functional RNA elements are poorly understood. The finding that lncRNA promoter sequences are often more conserved than the lncRNA genes that they regulate suggested the importance of lncRNA expression. Furthermore, although lncRNAs are expressed *on average* at a lower level than are protein-coding genes, they are often highly expressed in specific individual cells or under specific conditions. More importantly, a growing community of RNA biologists has documented a large variety of functions and mechanisms of action for a growing number of lncRNAs. LncRNAs can act across nuclear and cytoplasmic compartments—including their organelles and subcompartments, such as chromatin and polysomes—showing a variety of functions, including regulatory and structural functions. Existing evidence from studies in a broad range of cell types and tissues has indicated the nearly ubiquitous involvement of lncRNAs in almost every type of biological process studied. An amazing variety of fascinating mechanisms has been described over the years, with lncRNAs playing a central role in complexes with other RNAs, proteins, DNA, and even lipid membranes. Recently, the first catalytic lncRNA was discovered.

The field has moved a long way since its inception. After almost 20 years, we believe that this is an opportunity to review the extensive body of literature surrounding lncRNAs, starting from approaches to identify their expression and structural elements to delineating their biological functions and molecular mechanisms. This series of landmark reviews not only presents many exciting aspects of lncRNA structure and function but also provides a solid body of evidence documenting the critical biological roles played by this class of RNAs, which should convince any colleague who remains skeptical of the central role of lncRNAs in biology.

Starting with the census of lncRNAs, Lagarrigue et al. discuss the discovery of genes and the lncRNAs encoded by them. They also review the numbers of annotated lncRNAs across mammalian species. The human transcriptome is the most studied, with over 18,000 lncRNAs currently annotated in the latest release of the Gencode reference annotations, followed by the mouse, in which some 13,000 lncRNAs are currently annotated. The authors further discuss the discovery of lncRNAs in other mammalian species and the challenges presented by their high-sequence divergence and difficulties in broadly sampling tissues and cells. Of particular interest are discussions regarding the functional role of embedded repeat elements and the clinical importance of lncRNAs in human genetics as revealed by genome-wide association studies (GWAS). We agree that long-read sequencing (PacBio and Oxford Nanopore), particularly if this is coupled with single-cell approaches, will further expand the catalogs of cell-specific lncRNAs and the mRNAs that they may regulate.

Since their discovery, the expression of lncRNAs has been a longstanding issue of broad interest and debate, and their relatively low expression levels have been inferred to reflect a lack of function. While some lncRNAs are highly expressed in specific cell types, the majority are, on average, expressed at lower levels than are mRNAs, often at a rate that, when averaged across a cellular population, generates less than one copy per cell. The review of Grammatikakis and Lal addresses this: while there is general consensus that lncRNA molecules should be present at a certain concentration/number of molecules per cell, they note that by analyzing single-cell data, very often lncRNAs are highly expressed only in a subset of cells of a given tissue (for example, the brain), in a sub-population of induced pluripotent stem cells, or in a manner that is dependent on a specific cellular state (for example, during the cell cycle, in response to stress, etc.). Furthermore, these authors discuss the importance of cellular compartments. For instance, an lncRNA may be concentrated around regulatory regions or in one or more substructures in either the nucleus and/or cytoplasm of cells so as to manifest profoundly differing modalities of function relative to mRNAs, which are broadly engaged in the production of proteins.

Even known functional lncRNAs are often poorly evolutionarily conserved across species, despite conservation of their promoters and their 3′ processing sequences. To fully study lncRNAs across species, Ghanam et al. discuss the importance of creating transgenic mice that express the specific sequence of an lncRNA in its usual genomic context, so as to replace a region of the mouse genome with a fully humanized lncRNA locus. In particular, the authors show the power of this approach by studying an lncRNA expressed proximal to the *ACE2* gene, which encodes the SARS-CoV-2 cellular receptor.

Functions of lncRNAs may also reside in a small fraction of their transcribed sequence. This is surely the case for piRNAs, a class of ~ 29-nucleotide short RNAs that derive from lncRNA precursors and are largely implicated in suppressing transposable elements, regulating gene expression, imprinting genes, and antiviral defense. piRNAs also have a key role in fertility, as discussed by Sun et al., who detail the biogenesis of these fundamentally important small RNAs.

Final proof of function comes from directly assaying the consequence of knocking out or knocking down lncRNA gene expression. Genome-editing strategies for perturbation of lncRNAs are reviewed by Pulido-Quetglas and Johnson. They contrast the latest CRISPR–Cas genome-editing approaches with complementary siRNA and antisense oligonucleotide approaches. Specifically, this review focuses on the application of CRISPR–Cas to high-throughput functional screens, and the attendant requirements for design of bespoke targeting libraries and importance of accurate annotations of lncRNAs that this requires.

Camilleri-Robles et al. evaluate features of lncRNAs in *Drosophila* as compared to mammalian species (human and mouse). Although the majority of lncRNAs are not conserved in primary sequence, a fraction of them do show a clear pattern of sequence conservation. Furthermore, some lncRNAs in *Drosophila* contain region(s) of “microhomology” to potential mammalian homologs that are limited to relatively short stretches. More importantly, lncRNA function is often conserved. The authors discuss the chances that structure is the key feature for function, but structural studies are limited by the sparsity of available structures.

While conservation of primary sequence has been broadly discussed in relation to function, structure is likely to play a more fundamental role in lncRNA function. Sanbonmatsu also discusses how the accurate determination of lncRNA structures remains a huge challenge. Nuclear magnetic resonance is not suitable for determining the structure of very large RNAs. RNA structure should ideally be studied together with bound proteins; in addition, lncRNAs are often believed to be poorly structured. Her review reports examples of key lncRNA functions that depend on structure, highlighting the importance of additional studies to understand RNA structure.

Anderson and Anderson discuss the diverse roles of lncRNAs in heart development and diseases, reporting more than 25 lncRNAs that are known to be involved in the development and function of this organ. These lncRNAs are important regulators of processes as diverse as patterning during development, morphogenesis, and pathogenesis of the heart. Their regulatory mechanisms are varied and include molecular interactions through their sequences or secondary structures with DNA, proteins, and RNAs. While the identification of lncRNA involvement in human diseases provides a means to identify lncRNA function, the authors also discuss knock-down and knock-out technologies as ways to probe the functions of yet-to-be-characterized lncRNAs.

A large fraction of lncRNAs is confined to the nucleus, where they have specific regulatory or structural functions that are not yet fully understood. Kolpa et al. review the role of scaffold attachment factor A (SAF-A), a nuclear RNA-binding protein, and further characterize its role in binding lncRNAs to chromatin by providing data on several SAF-A variants. The authors identify a key role for CoT-1 RNAs, which represent the fraction of lncRNAs that is enriched for transcribed repeat elements. Results suggest that these RNAs function to maintain chromatin structure and prevent aberrant chromatin condensation.

Remaining in the nucleus but refocusing on nuclear organelles, Nakagawa et al. guide the reader through a fascinating world of various nuclear bodies, which are organized by a relatively well-characterized group of architectural RNAs (arcRNAs). There are several such nuclear bodies, and more than 20 lncRNAs that are involved. These lncRNAs include NEAT1 and its role in coordinating paraspeckle formation by forming specific folds and interacting with a series of RNA-binding proteins. Results suggest that more examples of this type of complex interaction are likely to exist in cells. Incidentally, as the name implies, arcRNAs can now be given a function and no longer need to be referred to based on what they do not do, i.e., “non-coding.”

The nucleolus is mostly engaged in rRNA biogenesis, although its composition is not fully understood. Hao and Prasanth review not only the role of rRNAs that are mostly transcribed from a group of acrocentric chromosomes, but also roles for a series of lncRNAs that originate from the spacer regions between rRNA genes. The transcription of these lncRNAs is often driven by RNA polymerase II and, thus, differs from transcription of the classic spacer regions. Some of these Pol II transcripts have important roles ranging from the regulation of rRNA gene transcription to regulation of the stress response. Surprisingly, many of these lncRNAs do not seem to initiate and terminate at unique sites. Defining the genomic positions of these diverse start and end sites and the full set of transcripts originating from them, along with the regulatory processes involved, await the results of single-molecule sequencing.

Last but not least is the crosstalk of lncRNAs during the host response to viral infections, as reviewed by Cazalla. This author discusses lncRNA interactions with the cellular network of the host and how they modify the cellular responses to infection. The article discusses in detail HUSUR1 and HUSUR2, which are produced by a herpesvirus, as well as lncRNAs of related function but likely independent evolution. These RNAs broadly interact with the machinery of host microRNAs, causing degradation, which ultimately modulates viral replication.

Altogether, this set of reviews convincingly discusses key specific aspects of lncRNA biology, placing these molecules in the central roles that they deserve. Given that the function of only a minor fraction of lncRNAs has been experimentally defined, we can foresee important discoveries of yet unknown and unexpected lncRNA functions, ranging from basic biological mechanisms to their role in health and disease. We can also foresee for the next decade an increased number of therapeutic applications, especially with the advent of antisense therapeutic strategies that are highly suitable for targeting lncRNAs. Given the importance of lncRNAs to regulating genes and genomic activities, these articles provide a state-of-the-art understanding of the lncRNA field, upon which future discoveries will be built.

